# Continuous recording of vital signs with a wearable device in pediatric patients undergoing chemotherapy for cancer—an operational feasibility study

**DOI:** 10.1007/s00520-021-06099-8

**Published:** 2021-03-03

**Authors:** Christa Koenig, Roland A. Ammann, Claudia E. Kuehni, Jochen Roessler, Eva Brack

**Affiliations:** 1 Pediatric Hematology/Oncology, Department of Pediatrics, Inselspital, Bern University Hospital, University of Bern, Bern, Switzerland; 2 Kinderaerzte KurWerk, Burgdorf, Switzerland; 3 Institute of Social and Preventive Medicine, University of Bern, Bern, Switzerland

**Keywords:** Continuous recording, Recording vital signs, Wearable device, Pediatric oncology, Supportive care

## Abstract

**Purpose:**

Pediatric patients with cancer are at high risk for severe infections. Infections can trigger changes of vital signs long before clinical symptoms arise. Continuous recording may detect such changes earlier than discrete measurements. We aimed to assess the feasibility of continuous recording of vital signs by a wearable device (WD) in pediatric patients undergoing chemotherapy for cancer.

**Methods:**

In this prospective, observational single-center study, pediatric patients under chemotherapy wore the Everion® WD for 14 days. The predefined patient-specific goal was heart rate recorded in good quality during ≥18/24 h per day, on ≥7 consecutive days. The predefined criterion to claim feasibility was ≥15/20 patients fulfilling this patient-specific goal.

**Results:**

Twenty patients were included (median age, 6 years; range, 2–16). Six patients aged 3–16 years fulfilled the patient-specific goal. Quality of heart rate recording was good during 3992 of 6576 (61%) hours studied and poor during 300 (5%) hours, and no data was recorded during 2284 (35%) hours. Eighteen of 20 participants indicated that this WD is acceptable to measure vital signs in children under chemotherapy.

**Conclusion:**

The predefined feasibility criterion was not fulfilled. This was mainly due to important compliance problems and independent of the WD itself. However, continuous recording of vital signs was possible across a very wide age range in pediatric patients undergoing chemotherapy for cancer. We recommend to study feasibility in the Everion® again, plus in further WDs, applying measures to enhance compliance.

**Trial registration:**

ClinicalTrials.gov (NCT04134429) on October 22, 2019.

**Supplementary Information:**

The online version contains supplementary material available at 10.1007/s00520-021-06099-8.

## Introduction

Pediatric patients with cancer and chemotherapy-induced neutropenia are at high risk of potentially life-threatening infections. Fever is often the only clinical detectable sign of such an infection. Fever in neutropenia is therefore treated as an emergency, leading to hospitalization, start of intravenous empirical broad-spectrum antibiotics, and close monitoring [1].

Delay of diagnosis and treatment may result in more intensive treatment, more adverse events, and increased mortality [2–4]. Longer time between hospital admission and start of antibiotics seems to be associated with worse outcomes [5]. Several clinical decision rules help to distinguish between severe and less severe infections [6–8], but with the exception of fever, they do not rely on vital signs.

Wearable devices (WDs) have become widely available and are used by athletes and nonathletes to monitor fitness and health indicators [9]. WDs are becoming smaller and more sophisticated and allow continuous monitoring of vital signs under any condition. In medical settings, noninvasive WDs are being introduced for continuous measurement of patients’ vital signs [10–12]. In adult oncology, studies have shown that data on physical activity levels, be it from consumer or research-grade WDs, can predict clinical outcomes [13]. But there is limited evidence for other vital signs, e.g., heart rate [13]. Continuous monitoring of vital signs has been shown to detect infections earlier than discrete measurements in specific settings. For example, heart rate variability drops several hours before clinical symptoms of sepsis are detectable in neonates [14] and in adults after bone marrow transplantation [15, 16]. In addition, recent studies showed that WDs are helpful for earlier detection of COVID-19 infections in adults [17–19]. WD-based continuous monitoring of vital signs may thus lead to shorter time to antibiotics and may help to distinguish patients at lower versus higher risk for severe infections. In pediatric patients with cancer, feasibility of continuous recording of temperature alone has recently been studied during hospitalization [20] and at home [21].

This study aimed to assess the feasibility of continuous multiparameter recording of vital signs in pediatric patients undergoing chemotherapy for cancer using a WD; to specifically assess feasibility in preschool patients; to compare continuously recorded vital signs with discrete measurements performed during clinical routine care; and to explore vital signs for specific patterns detectable before episodes of fever and infection.

## Methods

### Study design and participants

For this single-center observational pilot study, we consecutively screened patients aged between 1 month and 17.99 years. Patients were eligible if they were undergoing myelosuppressive chemotherapy for any malignancy, expected to last ≥1 month at time of recruitment; or at least one cycle of myeloablative therapy before autologous hematopoietic stem cell transplantation. Patients with local skin disease prohibiting wearing the WD were excluded. Patients, if able to judge, and their legal guardians gave written informed consent (IC) prior to study entry. All additionally gave written consent that the coded study data will be published. No financial or other compensation was given to patients or their parents. The study duration was 14 days per patient. The protocol had been registered at www.clinicaltrials.com (NCT04134429) and published in Protocol Exchange [22]. It was approved by the local Ethics Committees (Ethikkommission der Universitätskinderkliniken Bern, “Gesuch 1912”, Kantonale Ethikkommission Bern, BASEC-No.: 2019-01919) prior to patient recruitment. The study was conducted in accordance with the Declaration of Helsinki [23] and the principles of Good Clinical Practice.

### Wearable device and data management

**Fig. 1:**
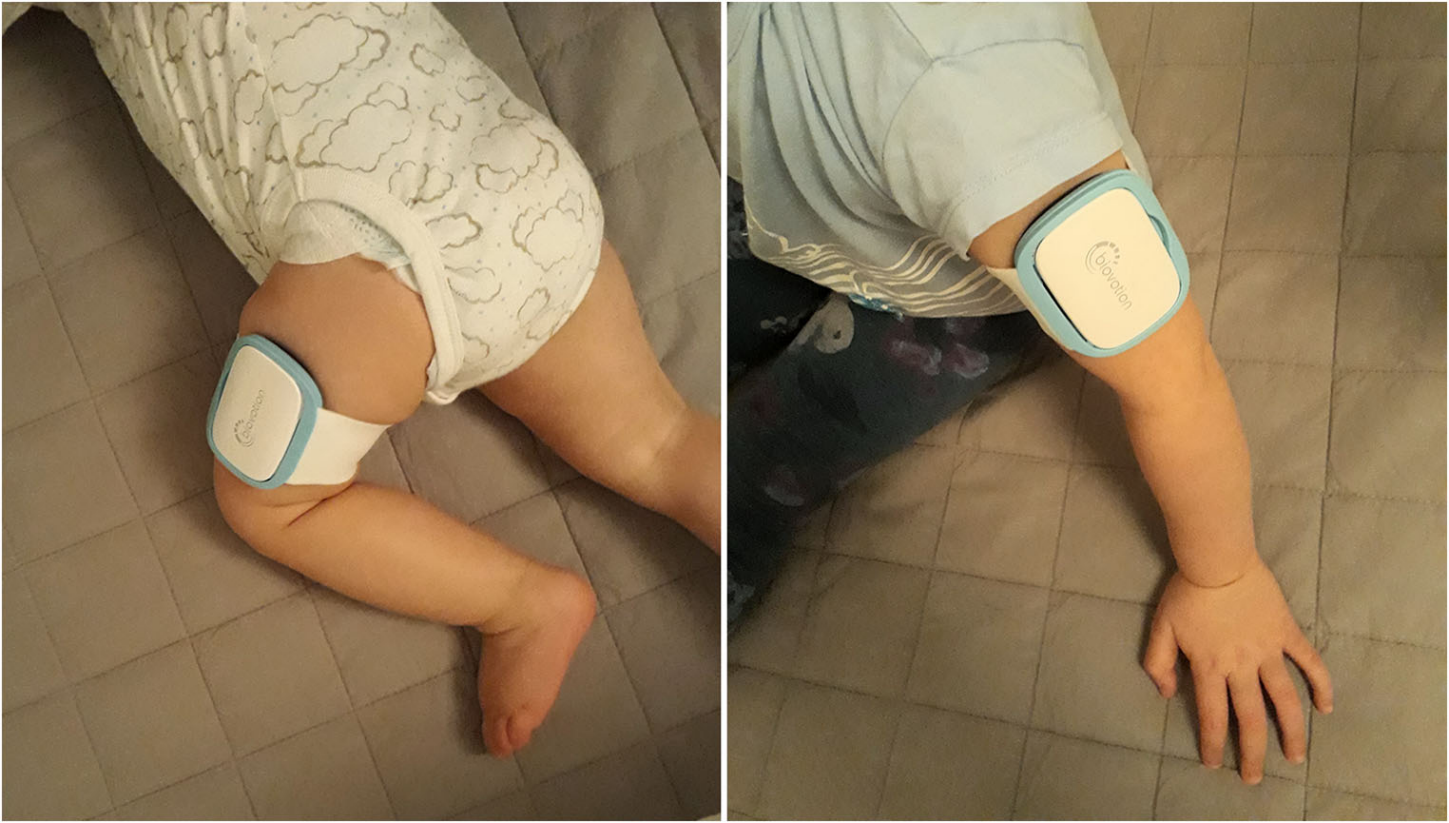
The Everion® device, placed on the upper leg of a 10-month-old and on the upper arm of a 3-year-old child. (Parents gave informed consent for publication of these photographs)

The WD used here was the Everion® VSM-1 device by Biovotion (now Biofourmis), Zurich, Switzerland (firmware version, 3.15.0) [24]. We chose this WD over other options, because it is light (approximately 40 g), without buttons and cables, can be placed at different body positions, and assigns quality scores to the main vital signs. Specifically, it is placed using elastic bands on the upper arm or, in small children, the upper leg and does not hamper a child during daily activities (Fig. 1). The elastic band cannot be tightened any further, what is more secure than an adjustable band for young children. The WD needs daily charging for approximately 2 h. It is waterproof but chlorine water and soap should be avoided. Therefore, we recommended to remove it for swimming or showering. No other study has used or validated this WD in children, and to avoid misinterpretation, participants and treating staff could not access the vital signs during the study. All vital signs were analyzed retrospectively.

This WD assigns a quality score (range 0 to 100) to six of nine vital signs recorded in this study: heart rate, heart rate variability (indicating heart beat to beat interval variation, calculated as root mean squared of successive differences of heart beats [25]), oxygen saturation, respiration rate, core temperature, and skin blood perfusion. No quality score is assigned to skin temperature, galvanic skin response (a measurement of electrodermal activity as an index of sympathetic nervous system activity [26, 27]), and health score (a score calculated from different vital signs claiming to indicate balance between bodily stress and recovery [28]).

For this study, only aggregates, calculated every 3 min from the primary measurements, performed every second, were used (Online Resource Text S1).

Aggregates were transferred to an Android^TM^ mobile phone application (AugmentStudy App, version 1.3.5) via Bluetooth®. The application displayed the connection status with the WD, battery status of the WD, and connection status with the Internet, be it via local wireless LAN or directly via the mobile phone network. Coded data was automatically uploaded by the application to a cloud-based password-protected dashboard (Everion Dashboard, version 6.0). Only the study team had access to this dashboard. The entire data processing (data in transport and data stored) was encrypted by Biovotion. For confidentiality, a data processing agreement was signed by Biovotion and the study center.

Data was retrieved by the researchers from the dashboard as device-specific json files using json scripts and then imported into R [29] via Microsoft® Excel. Data processing in R before analysis itself is described in Online Resource Text S2. After completion of analysis and storage of WD data in a local research database, data were irreversible deleted from the cloud-based dashboard. All non-WD data was collected on paper clinical research forms (CRFs) and stored using REDCap electronic data capture tools [30].

### Procedures

Participants, which could be parents or patients if applicable, were given a set of WD including size-specific elastic bands and charging station, a suitable mobile phone if needed, and a set of paper CRFs. They were asked that patients wear the WD as often as possible for the study period of 14 days. They were instructed in WD functions, handling and wearing, and how to fill out the daily CRFs on time of charging, connection with the application, physical activities, potential WD side effects, and results of optional discrete temperature measurements at home. They were encouraged to call the study center in case of problems or questions.

Daily data checks on the dashboard were done by one of the investigators (CK). Participants were contacted by phone in case of missing or implausible data. Possible reasons were discussed and instructions given, where applicable. During hospitalization or outpatient visits, calls were replaced by personal visits. Number, reason, type, duration of contacts, and actions taken were documented. Noncompliance, as not wearing or forgetting to charge the WD, did not lead to exclusion from the study. Within 3 days after the study period, a follow-up interview by phone, or if applicable during an outpatient visit, was conducted to assess acceptability and usability of the WD.

Information on discrete measurements of heart rate, oxygen saturation, respiration rate, and ear temperature, taken for clinical reasons during hospitalization or outpatient visits, was retrieved from patient charts.

### Outcomes

The primary outcome measure was heart rate quality score. The predefined patient-specific goal was heart rate recorded in good quality (quality score ≥50) during ≥18/24 h per day (midnight to midnight), during ≥7 consecutive days within the 14 days study period. The predefined criterion to claim feasibility was ≥15 of 20 patients (75%) fulfilling this patient-specific goal.

Secondary feasibility-related outcome measures were the quality scores of further vital signs (heart rate variability, oxygen saturation, respiration rate, core temperature, and skin blood perfusion), analyzed like the primary outcome measure, and the cumulative length of recorded vital signs per study day. These outcomes were additionally analyzed by skin type according to the Fitzpatrick scale [31] and by physical activity reported.

Additional feasibility-related outcomes were side effects, acceptability, reasons not to wear the WD, and effort for the investigators.

Further secondary outcomes were differences between discrete measurements and corresponding continuously recorded vital signs, plus specific vital sign patterns detectable within 48 h from fever (ear temperature of ≥39.0 °C, or ≥38.5 °C if fever is declared for clinical reasons [32]) or infection.

### Statistical analysis

As this is a feasibility study, a formal sample size calculation was not performed. A sample size of 20, able to identify ≥95% of usability problems, was chosen [33], including at least four patients below 6 years. Recorded data was categorized into three groups: good data quality (score, ≥50), poor quality (<50), and no data recorded. For comparison with discrete measurements, the corresponding means of vital sign aggregates recorded within 10 min were calculated, to account for imprecision of reported time points. For detection of specific patterns preceding fever or infection, vital signs were explored graphically. Version 3.6.0 of the R software [29] was used for analysis.

## Results

### Patients

From November 30, 2019, to January 13, 2020, 40 patients were consecutively screened for eligibility. Four did not meet inclusion criteria, 30 were asked for IC, and six were not asked after the predefined target sample size had been reached (Online Resource Figure S1). Twenty patients with a median age of 6 years (range, 2 to 16; 9 patients <6 years) were included in the study. Distribution of age, gender and type of malignancy of patients screened, refusing IC, not asked for IC, and those included in the study were comparable (Online Resource Table S1). In one patient, we realized only after start of the study that the remaining time of chemotherapy was below 1 month but covered the entire study duration of 14 days. This patient remained on study despite the formal violation of an inclusion criterion. One patient withdrew IC after eight study days, so a total of 274 days were analyzed. Patients were hospitalized during 33 days (12%), had outpatient visits on 13 days (5%), and remained at home during 228 days (83%). The WD was worn on the upper arm by 18 patients, on the wrist by one adolescent, and on the upper arm and the upper leg—though unsuccessfully in both locations because of repeated removal—by the youngest patient recruited.

### Primary outcome

Heart rate was recorded with good quality during 3992 of 6576 (61%) study hours. Quality was poor during 300 (5%) hours, and no data was recorded in the remaining 2284 (35%) hours (Table 1). Thus, quality was good during 3992 of 4292 (93%) hours with data recorded.

**Table 1:** Feasibility of recording vital signs in 20 pediatric patients with cancer, during the 274 days (6576 hours) of total study time^a^

	Patients fulfilling primary outcome^b^ [*n*] (%; 95% exact confidence interval)	Days with ≥ 18 h good data quality [days] (%)	Time with good data quality [hours] (%)	Time with poor data quality [hours] (%)
Heart rate	6 (30%; 12 to 54%)	142 (52%)	3992 (61%)	300 (5%)
Heart rate variability	1 (5%; 0 to 25%)	59 (22%)	3142 (48%)	1149 (17%)
Oxygen saturation	0 (0%; 0 to 17%)	3 (1%)	1103 (17%)	3189 (48%)
Respiration rate	4 (20%; 6 to 44%)	141 (51%)	4058 (62%)	233 (4%)
Core temperature	6 (30%; 12 to 54%)	140 (51%)	4037 (61%)	254 (4%)
Skin blood perfusion^c^	6 (30%, 12 to 54%)	142 (52%)	3992 (61%)	300 (5%)

^a^During 2284 hours (35%) no data was recorded

^b^Defined as good quality of the recorded vital sing during ≥18 hours per day on ≥7 consecutive days

^c^Skin blood perfusion has the same quality index as heart rate; results are therefore congruent

**Fig. 2:**
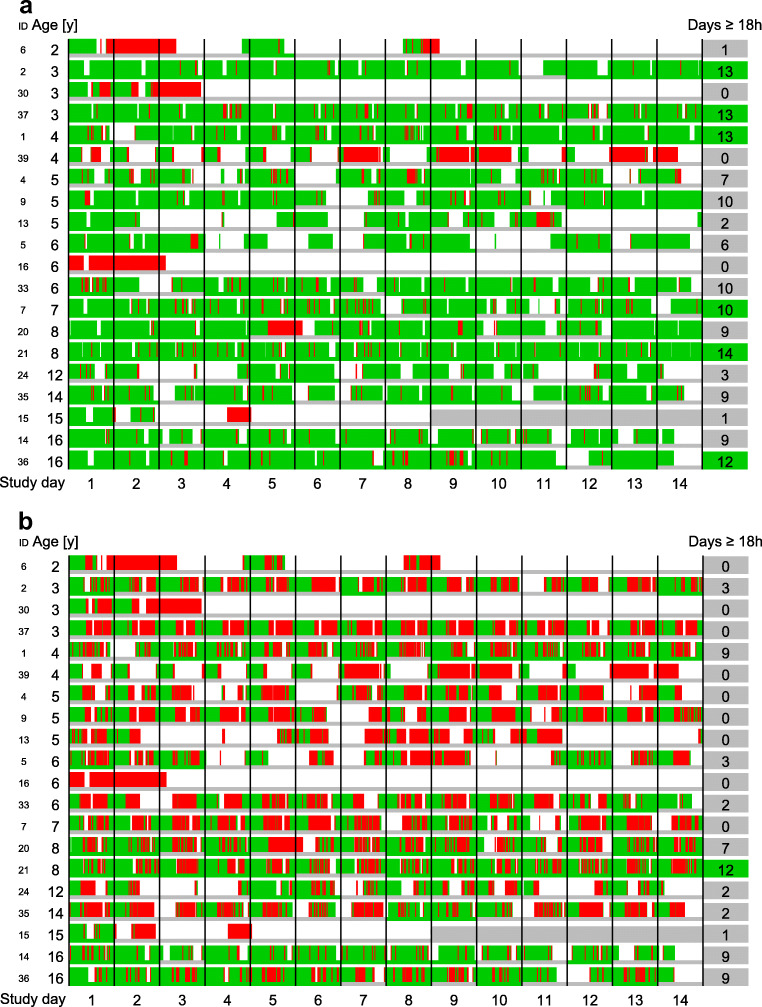
**a** Primary outcome measure—recording of heart rate. **b** Recording of heart rate variability. *Legend: green, good data quality; red, poor data quality; white, no data recorded; gray, no study days; days ≥18 h, number of days with at least 18 hours of good data quality per patient; ID, patient ID*

Six of 20 patients (30%; 95% confidence interval (CI) 12 to 54%), aged from 3 to 16 years, fulfilled the predefined patient-specific goal, i.e., heart rate recorded with good quality for ≥18/24 hours per day during ≥7 consecutive days (Table 1, Fig. 2a). Five additional patients fulfilled the goal, except that the ≥7 days were not consecutive. On 142 of 274 days (52%; 95% CI 46 to 59%), heart rate was recorded in good quality on ≥18/24 hours per day.

### Secondary feasibility-related outcomes

Like for heart rate, data quality was good in nearly all time with data recorded for respiration rate (95%) and core temperature (94%). It was slightly lower for heart rate variability (73%) and clearly lower for oxygen saturation (26%) (Tables 1 and 2, Fig. 2b, Online Resource Figure S2–S4).

**Table 2:** Vital sign recording in pediatric patients with cancer, per skin type^a^ and during physical activity

	Time with recorded data [hours] (%)	Patients [*n*]	Heart rate/skin blood perfusion^b^ [hours] (%)	Heart rate variability [hours] (%)	Oxygen saturation [hours] (%)	Respiration rate [hours] (%)	Core temperature [hours] (%)
Good data quality in							
All patients	4292 (100%)	20	3992 (93%)	3142 (73%)	1103 (26%)	4058 (95%)	4037 (94%)
Skin type I + II	2079 (100%)	11	1813 (87%)	1404 (68%)	530 (25%)	1925 (93%)	1930 (93%)
Skin type III	1988 (100%)	7	1967 (99%)	1567 (79%)	499 (25%)	1927 (97%)	1890 (95%)
Skin type IV + V	225 (100%)	2	211 (94%)	171 (76%)	74 (33%)	206 (92%)	218 (97%)
Good data quality during							
Physical activity reported	25.3 (100%)	7	24.2 (96%)	7 (28%)	1.1 (4%)	19.8 (78%)	21.2 (84%)
No physical activity reported	1852 (100%)	7	1818 (98%)	1472 (79%)	664 (37%)	1814 (98%)	1784 (96%)

^a^According to the Fitzpatrick skin type scale (reference [31])

^b^Skin blood perfusion has the same quality index as heart rate; the results are therefore congruent

**Fig. 3:**
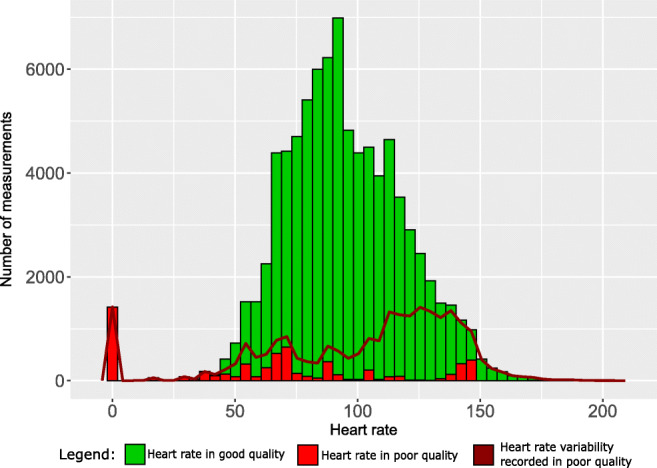
Histogram and quality assignment of heart rate, plus proportion of time with poor quality assignment of heart rate variability according to heart rate

**Fig. 4:**
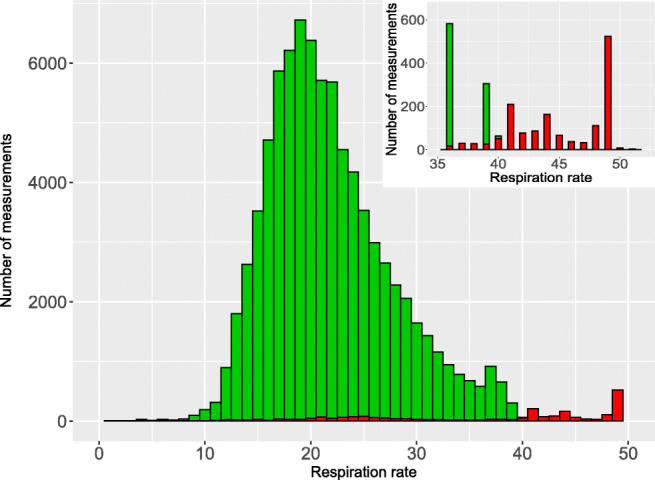
Histogram and quality assignment of respiration rate. *Legend: green, good data quality; red, poor data quality*

**Fig. 5:**
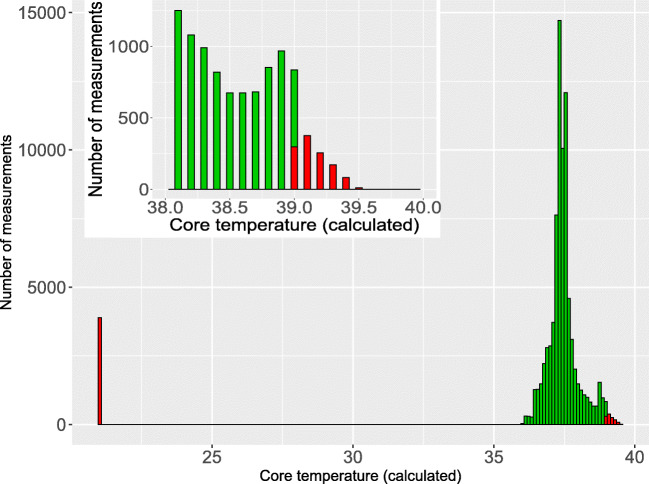
Histogram and quality assignment of core temperature (calculated). *Legend: green, good data quality; red, poor data quality*

Quality assignment was usually plausible, with two types of exceptions. First, the WD sometimes assigned good quality to an incorrectly measured vital sign. This was most obvious for a 6-year-old child (patient 16) who did not wear the WD at all. Though during the first 2 days the WD was switched on and recorded pseudo-vital signs in the box, assigning good quality to respiration rate and core temperature (Online Resource Figure S3 and S4). Second, the WD systematically assigned poor quality to vital signs at extremes, despite the fact that the distributions of these measurements looked plausible on the corresponding histograms. Specifically, this was the case for heart rates <40 beats per minute, for respiration rates >40 per minute, and for core temperatures >39.0 °C (Figs. 3, 4, 5).

Additionally, heart rate variabilities at heart rates >150 beats per minute were always computed to be 0, though correctly assigned to be of poor quality (Fig. 3, Online Resource Figure S5).

In different skin types, time with good data quality was comparable for all vital signs (Table 2).

Eight patients (median age, 7 years; range, 3 to 16) reported 56 episodes of physical activities. Of these, six were no physical activities as such (e.g., “vomiting”), during two the WD was not worn and for two the end time was unknown. The remaining 46 physical activities were analyzed. Data quality during versus outside physical activities was comparable for heart rate, but inferior for heart rate variability, oxygen saturation, respiration rate, and core temperature (Table 2).

### Side effects

One patient aged 5 years developed a superficial skin lesion underneath the device after scratching due to itching on study day 11, because he had refused to change the WD position despite red and itchy skin. The skin healed rapidly after changing the position. Further side effects reported were irritated skin (*n*=4), itching (*n*=3), and sweating (*n*=7).

### Acceptability

A reason for not wearing the WD was reported for only 14 of 85 days in which the device was not worn for ≥12 hours: forgetting (*n*=4) or refusing (*n*=2) or inconvenience (*n*=2) to wear the WD, MRI examination (*n*=2), swimming (*n*=3), and need for charging (*n*=1).

Participants judged the device to be wearable on 116 (42%) of 274 study days, not to be wearable on 26 (9%) days, with missing answers on 132 (48%) days.

The follow-up interview was done with patients themselves in the four oldest patients (14 to 16 years) and with parents for the remaining patients. At follow-up, most participants considered the Everion® to be a suitable device to record vital signs in children undergoing chemotherapy for cancer (*n*=18), to be easy (*n*=14), and comfortable to wear (8 yes; 8 sometimes; 3 no; 1 unable to decide). Only few reported problems wearing the WD (3 yes, 3 sometimes, 14 no). Some reported the WD to be too big (*n*=5) or to slip occasionally (*n*=3). Nobody judged the WD as too heavy (*n*=0). Some participants reported problems with charging the WD (*n*=3), with connecting (*n*=5) and with using (*n*=1) the mobile phone application, and with loosening of the elastic band (*n*=2). All technical problems were reported by parents and none by the adolescent patients answering to the follow-up questions themselves. The necessity to repeatedly restart the mobile phone application because of connection failure was reported as demotivating.

### Effort for the investigators

Inclusion and instruction took approximately 2 h per patient (total, 40 h). Daily data availability checks took about 5 min per patient and study day (total, 23 h). On 42 days, 52 contacts for problems with 15 participants were needed, with a maximum of 6 contacts per participant. The cumulative duration of these contacts was 390 min (6 h), and 43 (83%) contacts were initiated by the investigators. The reason for such contacts were missing data (*n*=28), poor data quality (*n*=3), and other reasons (*n*=9). The most common problems identified were non-wearing of the device (*n*=18) and technical problems (data transfer problems, *n*=13; other technical problems, *n*=5).

### Comparison of discrete measurements and continuously recorded vital signs

The analysis of differences between discrete measurements and continuously recorded aggregates of vital signs, as defined in the protocol, was found to yield massively distorted results because rapidly changing discretely measured vital signs were compared to aggregated means covering up to 20 min. We deliberately refrain from displaying these non-meaningful results here.

### Exploration for specific patterns preceding infections.

Three episodes with fever (one in neutropenia) and one local skin infection were recorded in three different patients (5, 9, 13; aged 5 to 6 years). During all episodes, the patients were hospitalized. Visual exploration of the recorded vital signs within 48 h preceding these episodes was hampered by missing data and did not allow to detect a potential pattern (Online Resource Figure S11-S20).

## Discussion

The predefined criterion to claim feasibility was clearly missed. Only six instead of ≥15 among 20 patients fulfilled the patient-specific goal of the primary outcome measure. This was mainly due to missing data because the WD was not worn, i.e., low compliance. We see four relevant and potentially remediable factors leading to low compliance: First, there was no direct benefit of study participation for parents and patients; this may be different during interventional trials. Second, there was no feedback on quality of recording and communication with the application; such feedback can be implemented in an updated application. Third, demotivating technical problems mainly regarding communication can as well be solved using updated hardware or software. Finally, the only side effects reported were local skin irritations; regularly changing the wearing site can prevent them.

This negative main result contrasts with two encouraging findings: The vast majority of participants considered the WD to be suitable, easy, and comfortable to wear. And the age range of patients fulfilling the patient-specific goal was wide, from 3 to 16 years. This implies that potential future clinical applications of WDs for continuous and at home recording of vital signs can be envisaged from preschool children to adolescents. Infants were not studied here.

In healthy children, continuous monitoring has been reported for heart rate [34] and physical activity [35] with wrist-worn WDs. Good compliance for wrist-worn WDs has been reported in a large study (*n*=886), but the minimal wear time criterion of ≥10 h per day had been much lower than here [36]. In children undergoing chemotherapy for cancer, studies have reported on physical activity assessment by WDs worn on the ankle [37], waist [38, 39], or clothes [40], and two small pilot studies have reported on continuous monitoring of temperature with patches in hospitalized patients [20] and at home [21]. Continuous recording of vital signs beyond temperature and activity using WDs has not yet been reported in this patient group.

The Everion®, the device used here, has been reported to be comfortable during day- and nighttime without displacement in a study of 115 adult patients with epilepsy [41]. Uninterrupted recording is essential for prediction of imminent fever or infections and requires that the WD is comfortable, remains in place, and does not hamper the child’s daily activities. In patients with good compliance, these requirements seemed to be fulfilled in our study.

In contrast to most other WDs, assignment of quality scores to recorded vital signs is routinely implemented in the Everion®. These scores were useful to exclude many non-plausible results from analysis. Non-plausible poor quality was systematically assigned to part of the vital signs in very low or very high ranges, which are physiological for children, however. This issue, which may distort the function of pattern search algorithms, may be due to the use of aggregates. Should it remain unchanged when non-aggregated date are used, it might be solved together with the implementation of off-body detection by updating the firmware without changing the hardware.

The study has several limitations. (i) The WDs were studied for only 14 days and compliance might be different during long-term use. (ii) The small number of patients precluded an analysis of patient factors influencing the primary outcome, limits the validity of the skin type assessment, and allowed only rudimentary exploration for specific patterns preceding episodes of fever and infection. (iii) The planned comparison of discrete measurements versus continuously was not feasible because the exact second-wise time point of discrete measurements was not available.

The major strength of this study is that the patients included represent an unbiased sample of children undergoing chemotherapy for cancer from 2 to 16 years of age. The broad assessment of feasibility including parents’ and patients’ opinion and effort for the investigator allows a comprehensive judgment on feasibility in pediatric patients undergoing chemotherapy for cancer. The problems identified and described above can be addressed in future studies

In conclusion, in the configuration studied, the predefined feasibility criterion was not fulfilled, due to important compliance problems. These are essentially independent of the WD itself and correspondingly may be resolved in future studies. However, we found that continuous recording of vital signs by the Everion® WD is feasible across a very wide age range in pediatric patients undergoing chemotherapy for cancer. The results of this study will influence the design of future WD studies including those aiming to identify patterns predicting fever or infection. Specifically, we recommend to study feasibility in the Everion® again, plus in further WDs, applying measures to enhance compliance.

## Supplementary Information

ESM 1 (PDF 3.06 mb)

## Data Availability

The data that support the findings of this study will be made openly available in figshare at 10.6084/m9.figshare.13471338.v6.
