# Supplementary material for: Continuous recording of vital signs with a wearable device in pediatric patients undergoing chemotherapy for cancer—an operational feasibility study
Source: Support Care Cancer. 2021 Mar 3;29(9):5283–92. doi: 10.1007/s00520-021-06099-8 (PMC7925259; doi:10.1007/s00520-021-06099-8)

Supplementary material

Continuous recording of vital signs with a wearable device in pediatric patients undergoing chemotherapy for cancer – an operational feasibility study

Christa Koenig*, Roland A. Ammann*, Claudia E. Kuehni, Jochen Roessler, Eva Brack

*shared first authorship

Online Resource Text S1 - Calculation of aggregate results and aggregate quality assignments 2
Online Resource Text S2 - Data processing in R 2
Online Resource Figure S1 - Flow Chart Patient recruitment 4
Online Resource Figure S2 – Recording of oxygen saturation 5
Online Resource Figure S3 – Recording of respiration rate 5
Online Resource Figure S4 – Recording of core temperature 6
Online Resource Figure S5 – Histogram of recorded heart rate variability 6
Online Resource Figure S6 – Histogram of recorded oxygen saturation 7
Online Resource Figure S7 – Histogram of recorded skin temperature 7
Online Resource Figure S8 – Histogram of recorded skin blood perfusion 8
Online Resource Figure S9 – Histogram of recorded galvanic skin response (GSR) 8
Online Resource Figure S10 – Histogram of recorded health score 9
Online Resource Figure S11 – Heart rate, all patients 10
Online Resource Figure S12 – Heart rate variability, all patients 11
Online Resource Figure S13 – Oxygen saturation, all patients 12
Online Resource Figure S14 – Respiration rate, all patients 13
Online Resource Figure S15 – Core temperature, all patients 14
Online Resource Figure S16 – Skin temperature, all patients 15
Online Resource Figure S17 – Skin blood perfusion, all patients 16
Online Resource Figure S18 – Galvanic skin response, all patients 17
Online Resource Figure S19 – Health score, all patients 18
Online Resource Figure S20 – Exploration of heart rate variability within fever or infection 19

Online Resource Text S1 - Calculation of aggregate results and aggregate quality assignments

For the six vital signs with a quality score assigned (heart rate, heart rate variability, oxygen saturation, respiration rate, core temperature and skin blood perfusion) the aggregate measurement over the three past minutes is a quality-weighted average of the corresponding 180 primary per-second measurements. All primary measurements with a quality score >50 are used and then weighted by subtracting 30 of the assigned quality score. E.g., a measurement with a quality score of 40 is not used for calculating the aggregate, a measurement with a quality score of 50 is used with a weight of 20, and a measurement with a quality score of 90 is used with a weight of 60.

For the three vital signs without quality score assignment, the calculation is different: For skin temperature and galvanic skin response, the aggregate is the median of all primary measurements within the three past minutes. For health score, the last measurement is used as aggregate.

Aggregate quality assignments are the average of all quality assignments >50, if more than 10% of quality assignments are >50. Otherwise it is the average of all quality assignments <50. (Data Glossary by Biovotion, revision 1, generation date: 28-Mar-2019, unpublished).

Online Resource Text S2 - Data processing in R

Before analysis, device-specific raw data files were processed into patient-specific restricted data files in five steps:

First, the information, always sorted incompletely, was sorted according to timestamps.

Second, double lines of information with identical timestamps were deleted (randomly distributed lines in all device files, 5 to 75 lines per device, corresponding to 0.1 to 1.2% of data).

Third, absolute dates of timestamps were replaced by study days (1 to 14) after adding 1 hour to the GMT (Greenwich Mean Time) based WD timestamps in order to correspond to CET (Central European Time) used for the study.

Fourth, device-specific data files was transferred into patient-specific data files, where applicable. Specifically, data files from devices used for different patients were split into different patient files. And data files from devices were merged for patients having used different devices.

Finally, variables not studied here plus information before and after the study period were deleted.

The resulting patient-specific data files thus contained the complete information for a single patient, restricted to variables studied here and to the study period of 14 days.

Online Resource Table S1 Basic patient characteristic.

	Patients screened	Patients who declined informed consent	Patients not asked for informed consent	Patients in study
Number of patients	40[a]	10	6	20
Age [years; median (range)]	6 (0 to 16)	6 (1 to 16)	7 (2 to 16)	6 (2 to 16)
<6 years	20	5	2	9
Sex				
Female	19	5	2	9
Male	21	5	4	11
Type of malignancy				
Acute lymphoblastic leukemia	20	5	3	12
Other hematologic malignancies	3	0	1	2
Central nervous system tumor	6	1	1	3
Solid tumor	11	4	1	3

[a] Of 40 patients screened, four did not meet inclusion criteria.

Online Resource Figure S1 - Flow Chart Patient recruitment

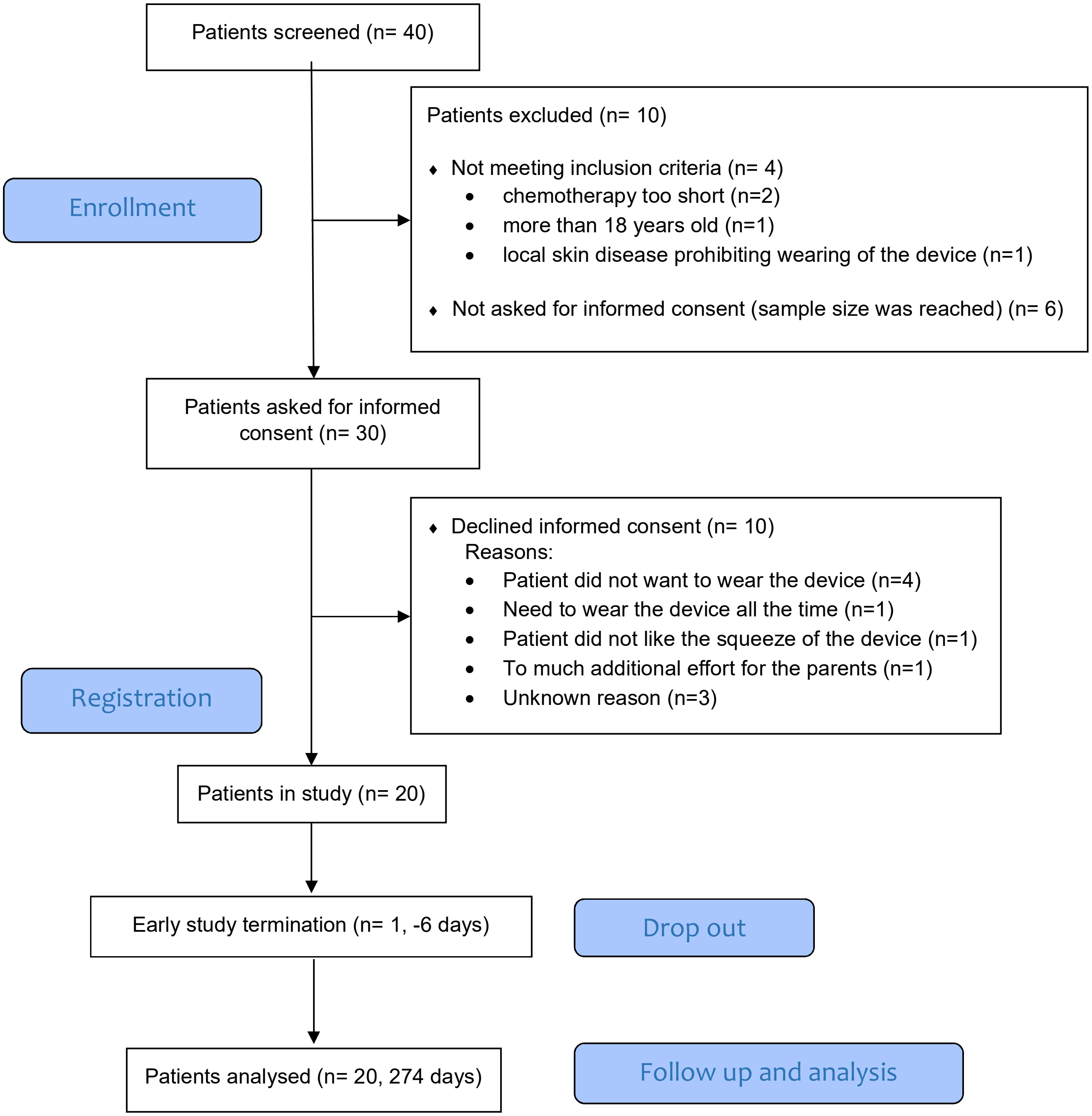

Online Resource Figure S2 – Recording of oxygen saturation

Green: good data quality; Red: poor data quality; White: no data recorded; Gray: no study days; Days ≥18h: number of days with at least 18 hours of good data quality per patient; ID: patient ID

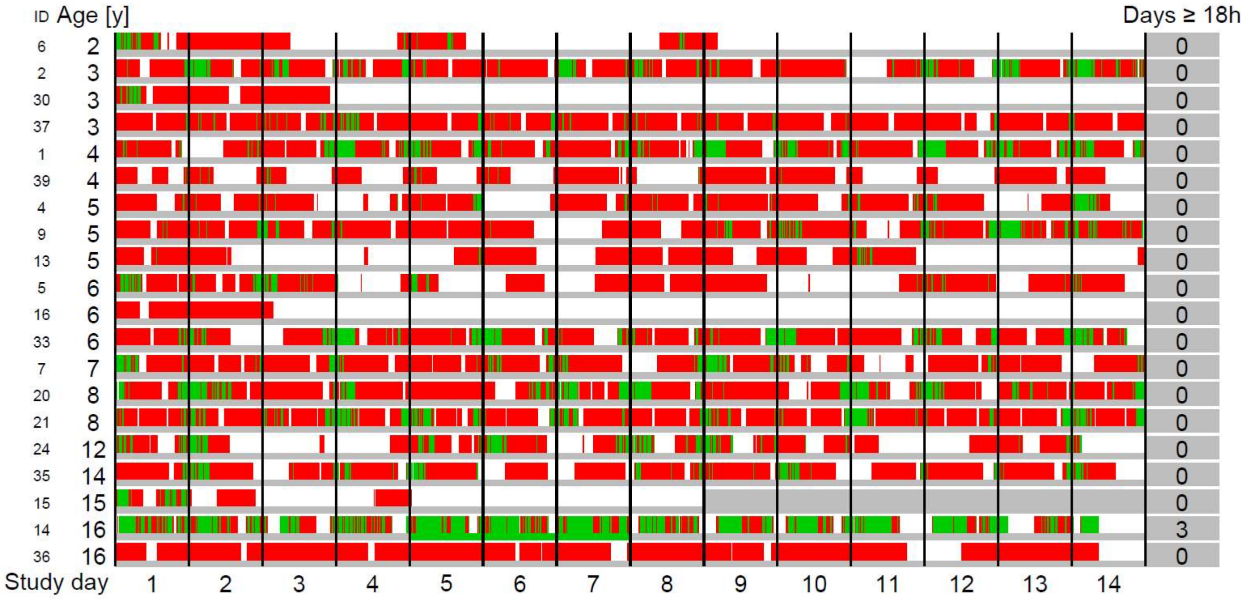

Online Resource Figure S3 – Recording of respiration rate

Green: good data quality; Red: poor data quality; White: no data recorded; Gray: no study days; Days ≥18h: number of days with at least 18 hours of good data quality per patient; ID: patient ID

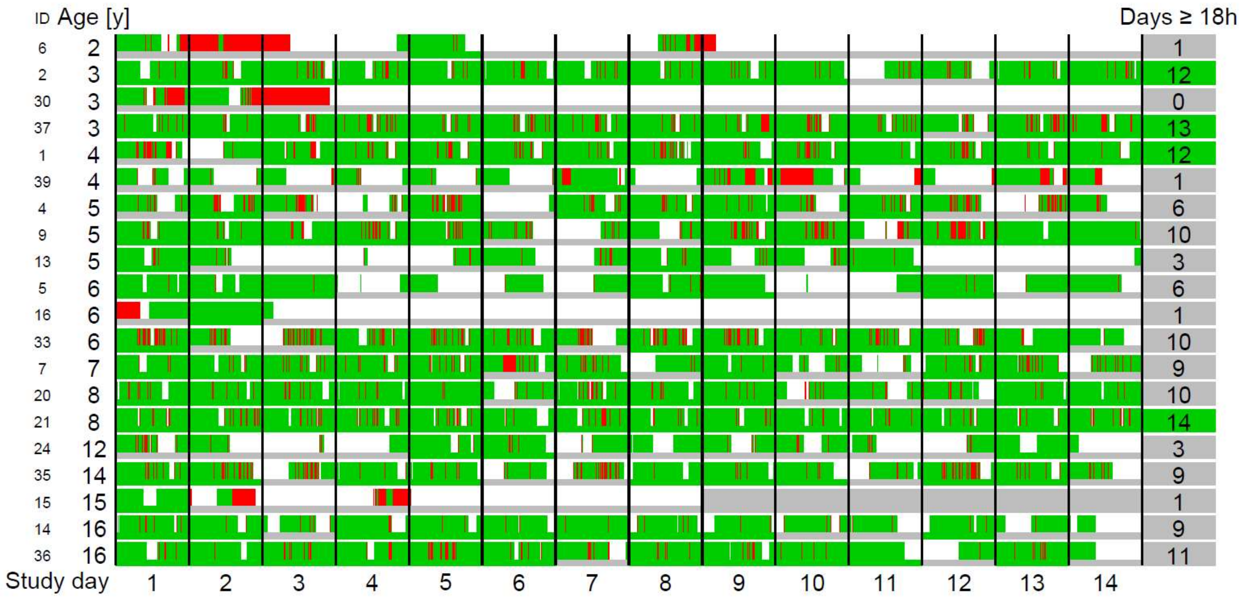

Online Resource Figure S4 – Recording of core temperature

Green: good data quality; Red: poor data quality; White: no data recorded; Gray: no study days; Days ≥18h: number of days with at least 18 hours of good data quality per patient; ID: patient ID

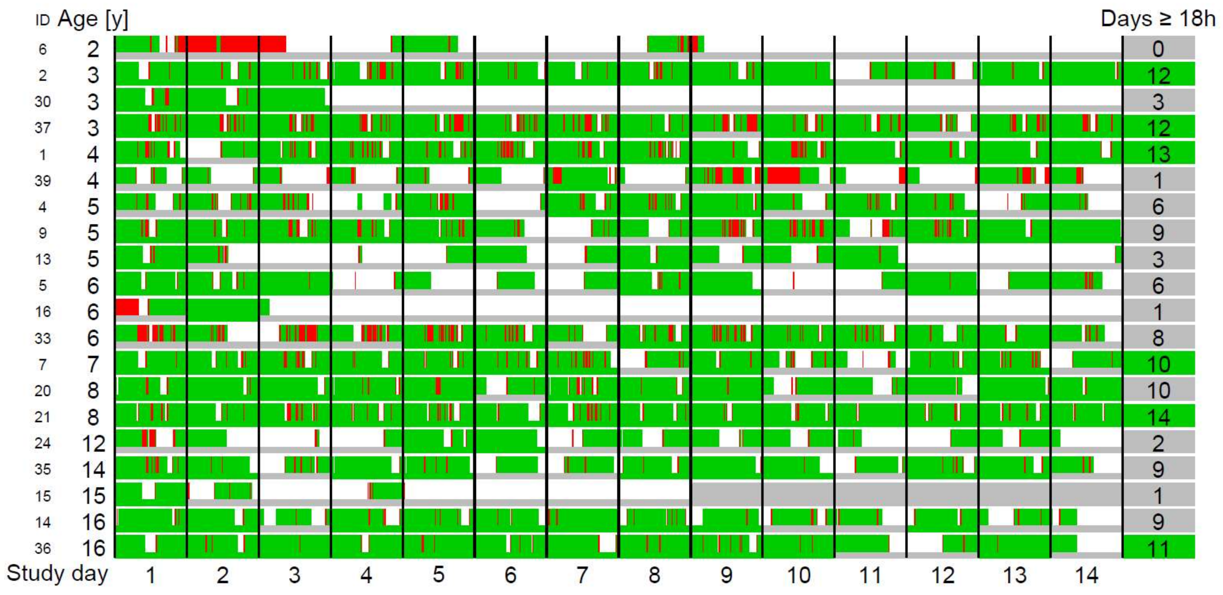

Online Resource Figure S5 – Histogram of recorded heart rate variability

Green: good data quality, red: poor data quality

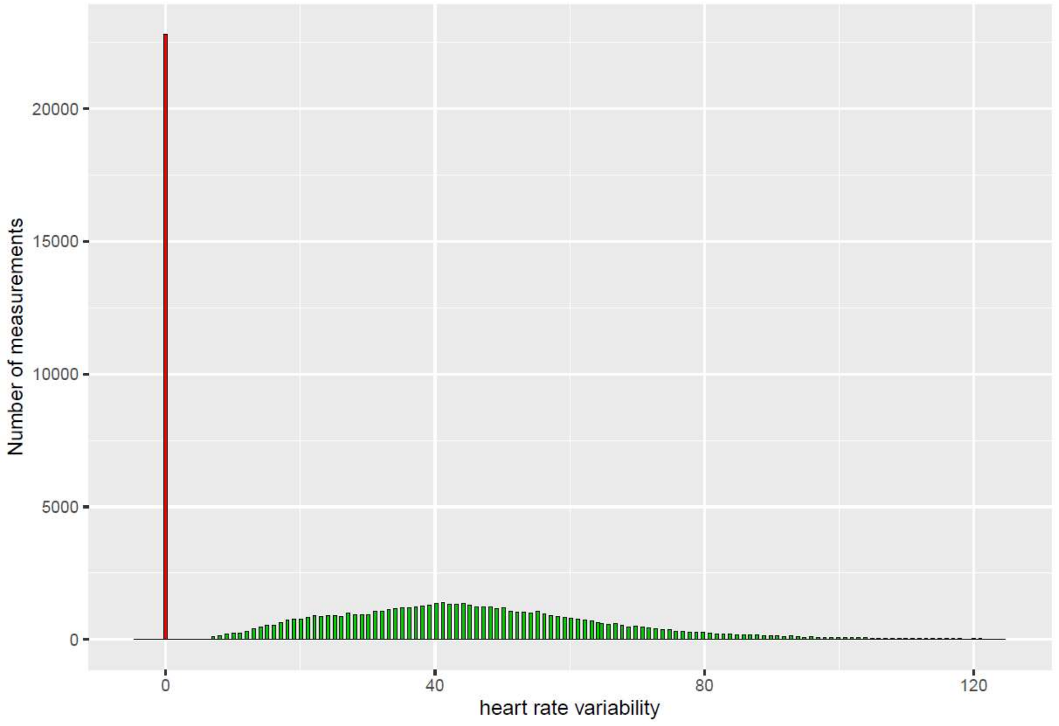

Online Resource Figure S6 – Histogram of recorded oxygen saturation

Green: good data quality, red: poor data quality

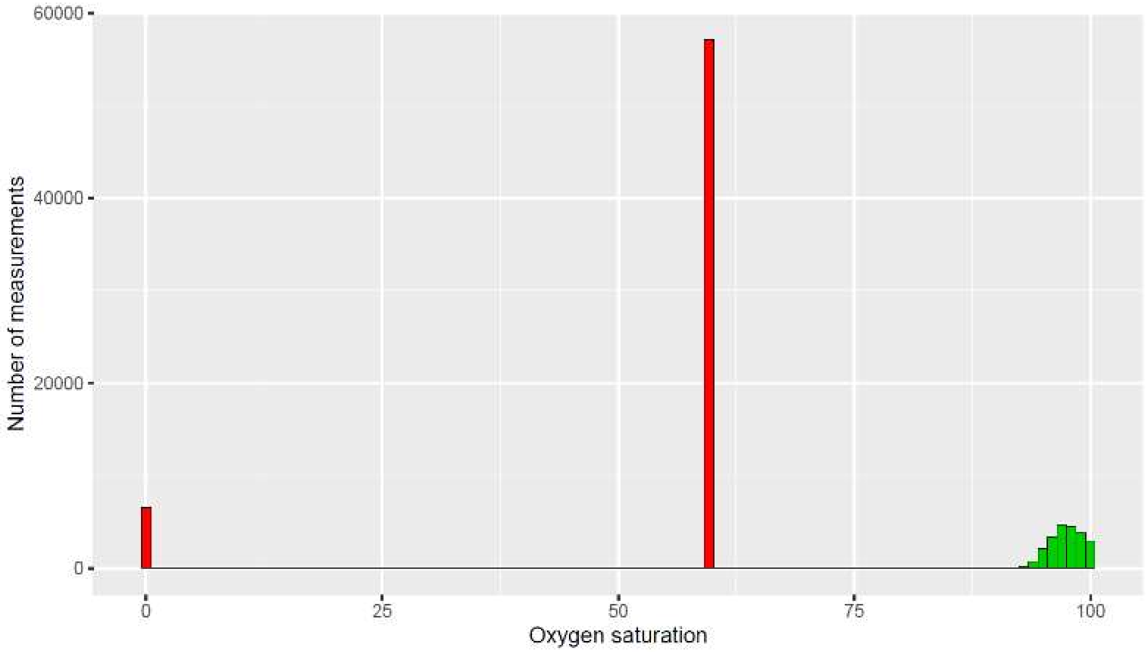

Online Resource Figure S7 – Histogram of recorded skin temperature

No quality assignment for skin temperature

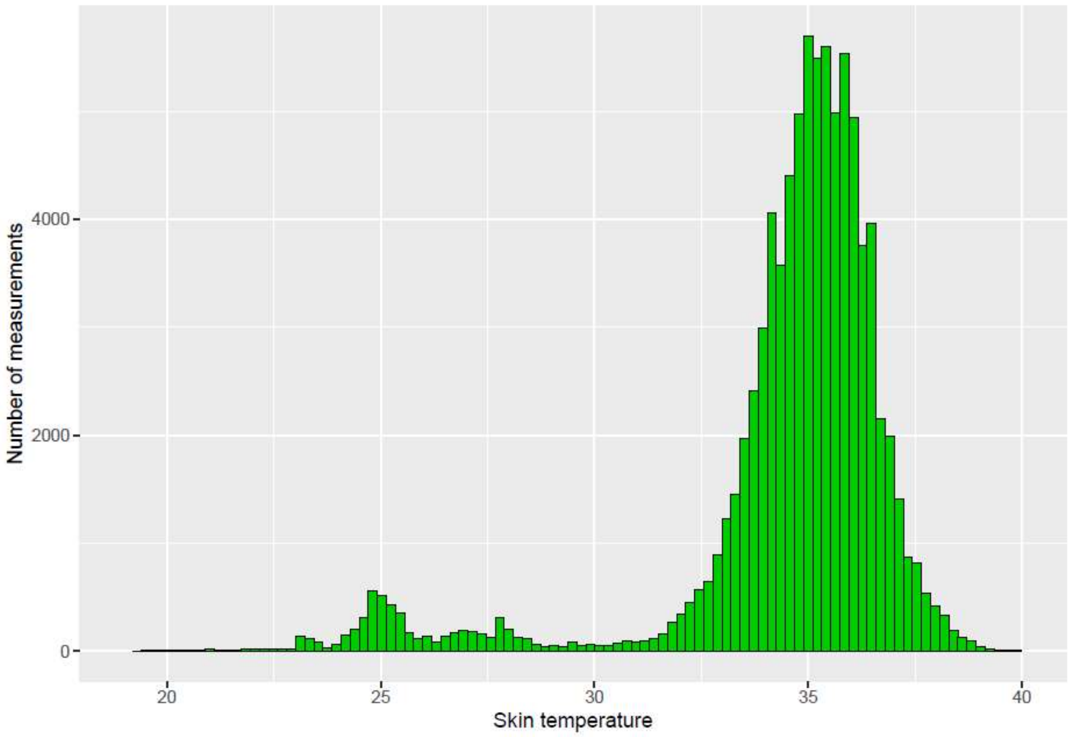

Online Resource Figure S8 – Histogram of recorded skin blood perfusion

Green: good data quality, red: poor data quality

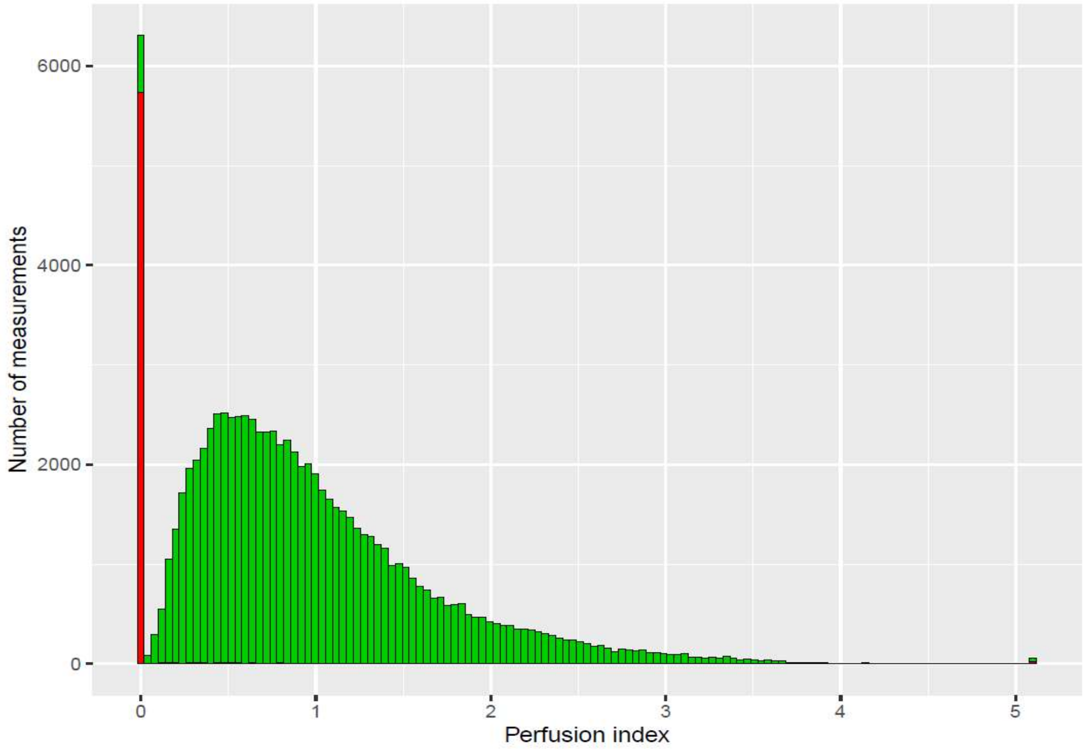

Online Resource Figure S9 – Histogram of recorded galvanic skin response (GSR)

No quality assignment for galvanic skin response

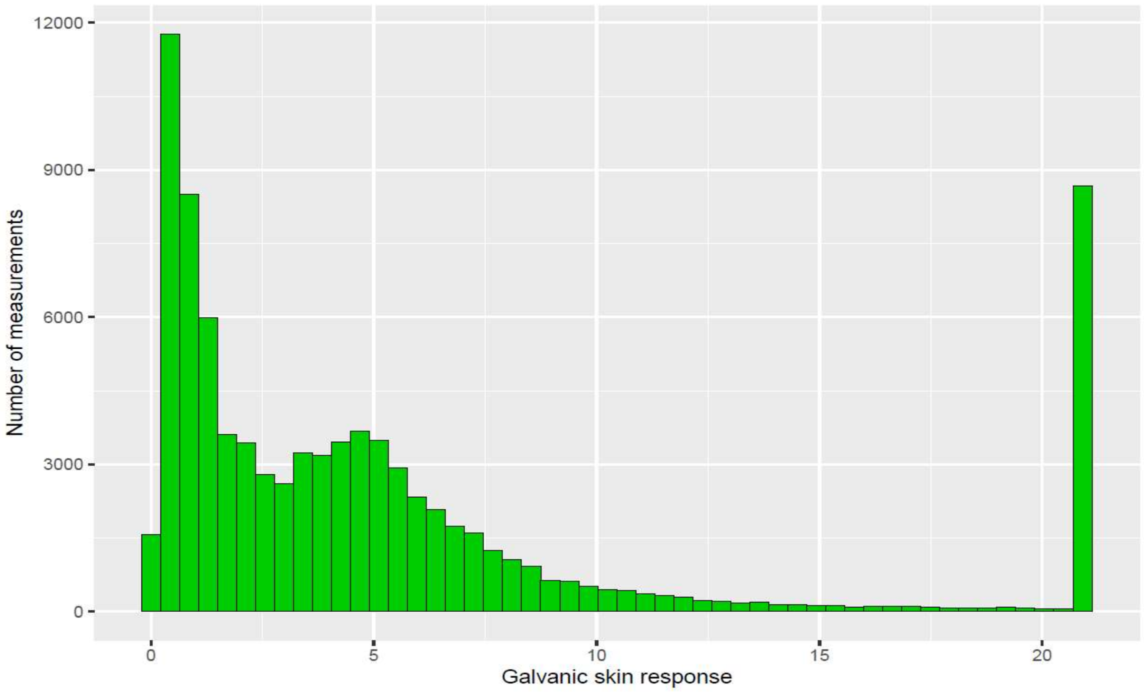

Online Resource Figure S10 – Histogram of recorded health score

No quality assignment for health

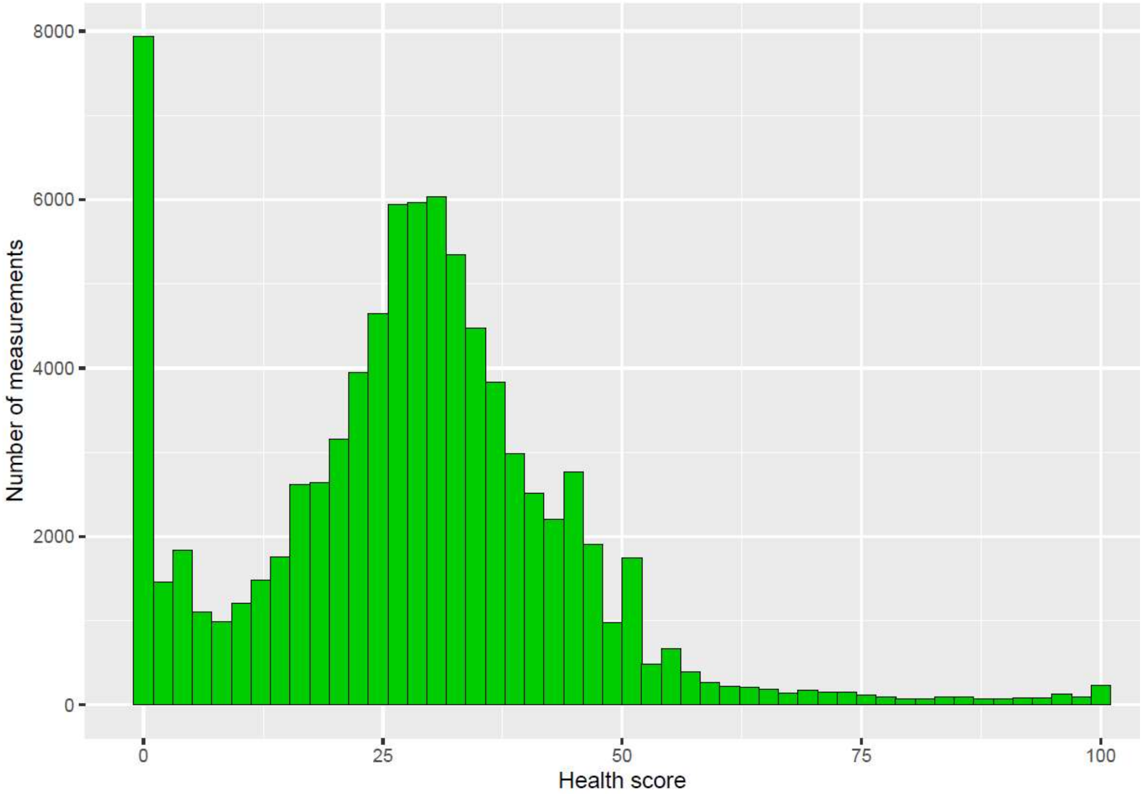

Online Resource Figure S11 – Heart rate, all patients

Recorded heart rate in good quality (score ≥50) in all patients during the 14 days study period. Red lines: event with fever or infection.

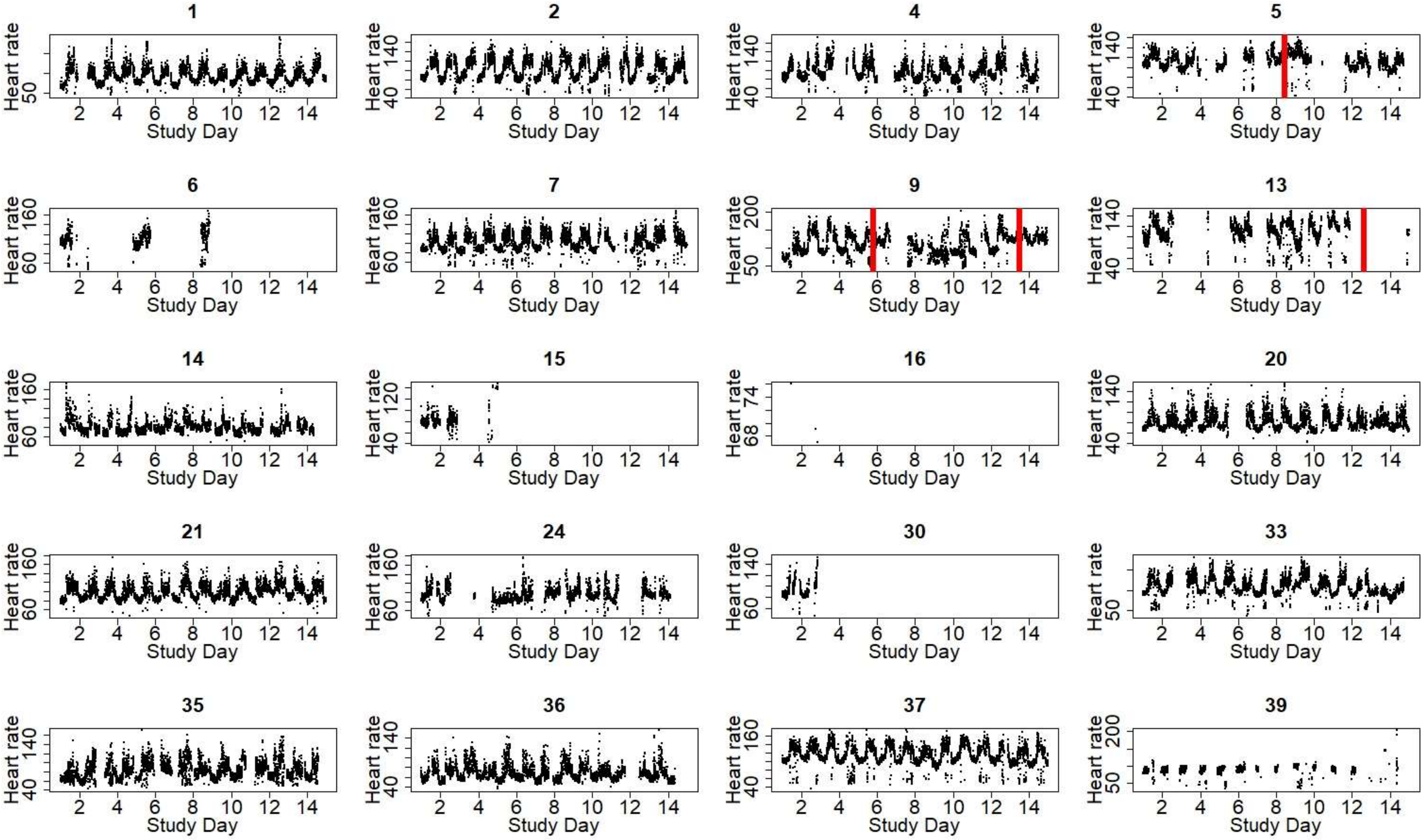

Online Resource Figure S12 – Heart rate variability, all patients

Recorded heart rate variability in good quality (score ≥50) in all patients during the 14 days study period. Red lines: event with fever or infection.

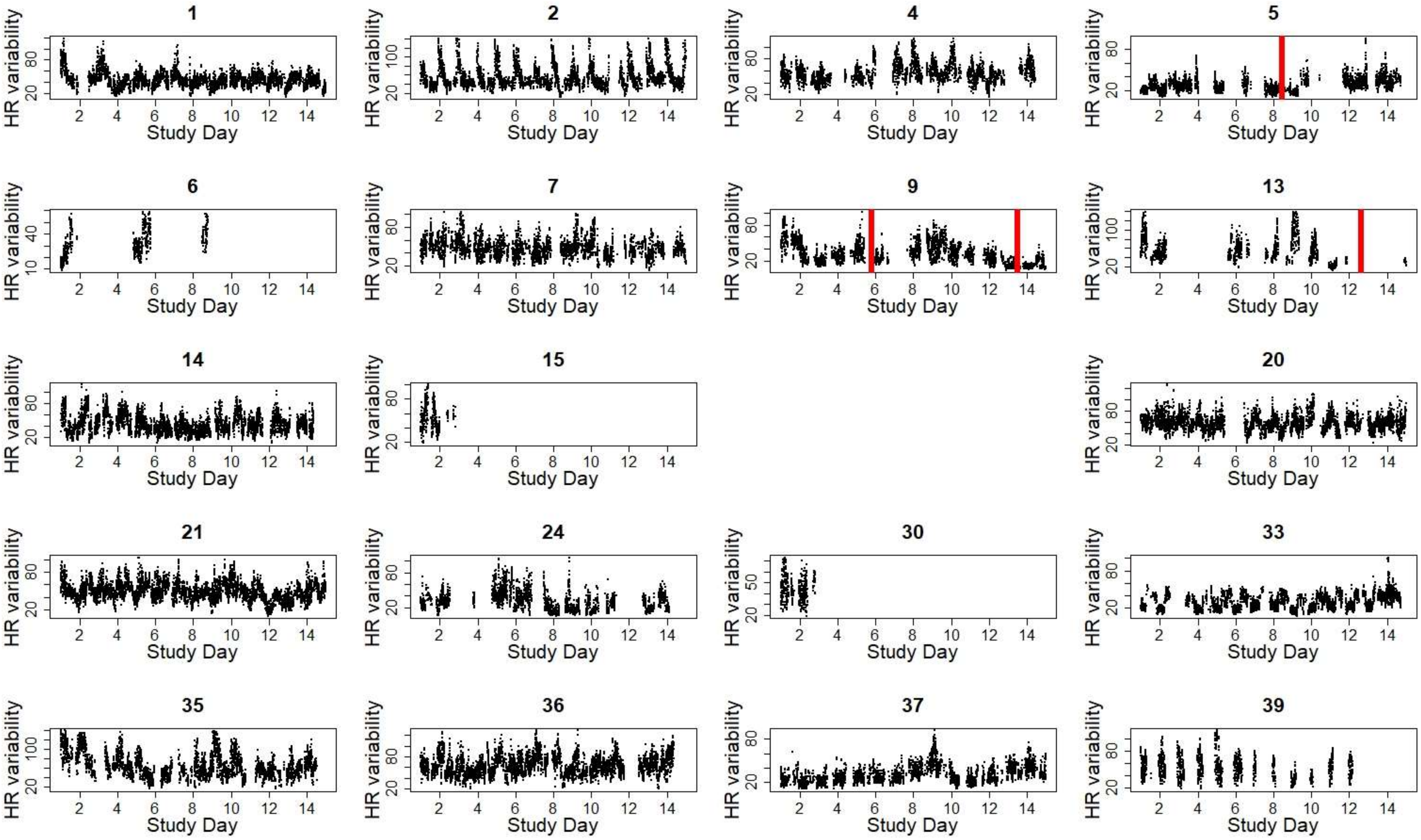

Online Resource Figure S13 – Oxygen saturation, all patients

Recorded oxygen saturation in good quality (score ≥50) in all patients during the 14 days study period. Red lines: event with fever or infection.

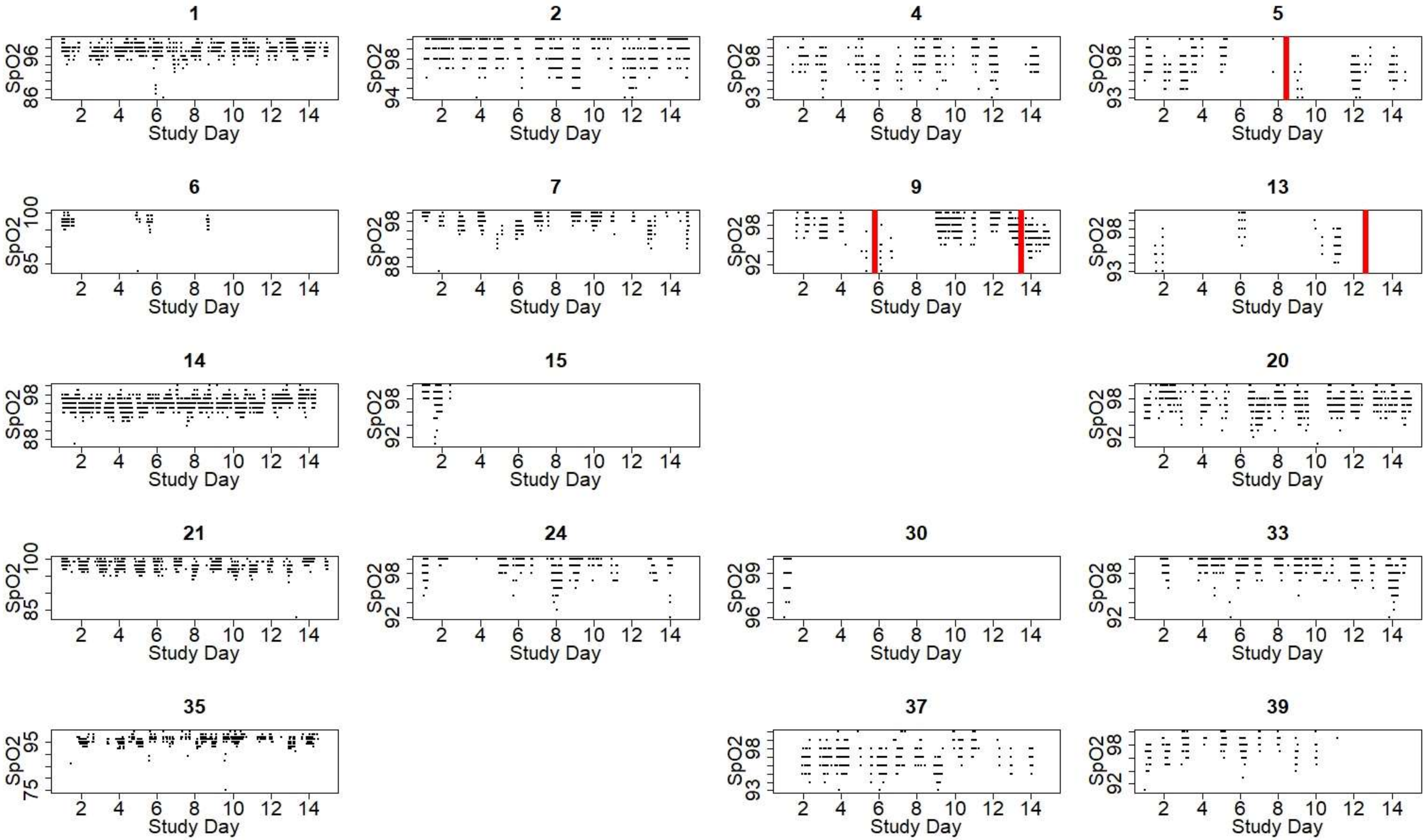

Online Resource Figure S14 – Respiration rate, all patients

Recorded respiration rate in good quality (score ≥50) in all patients during the 14 days study period. Red lines: event with fever or infection.

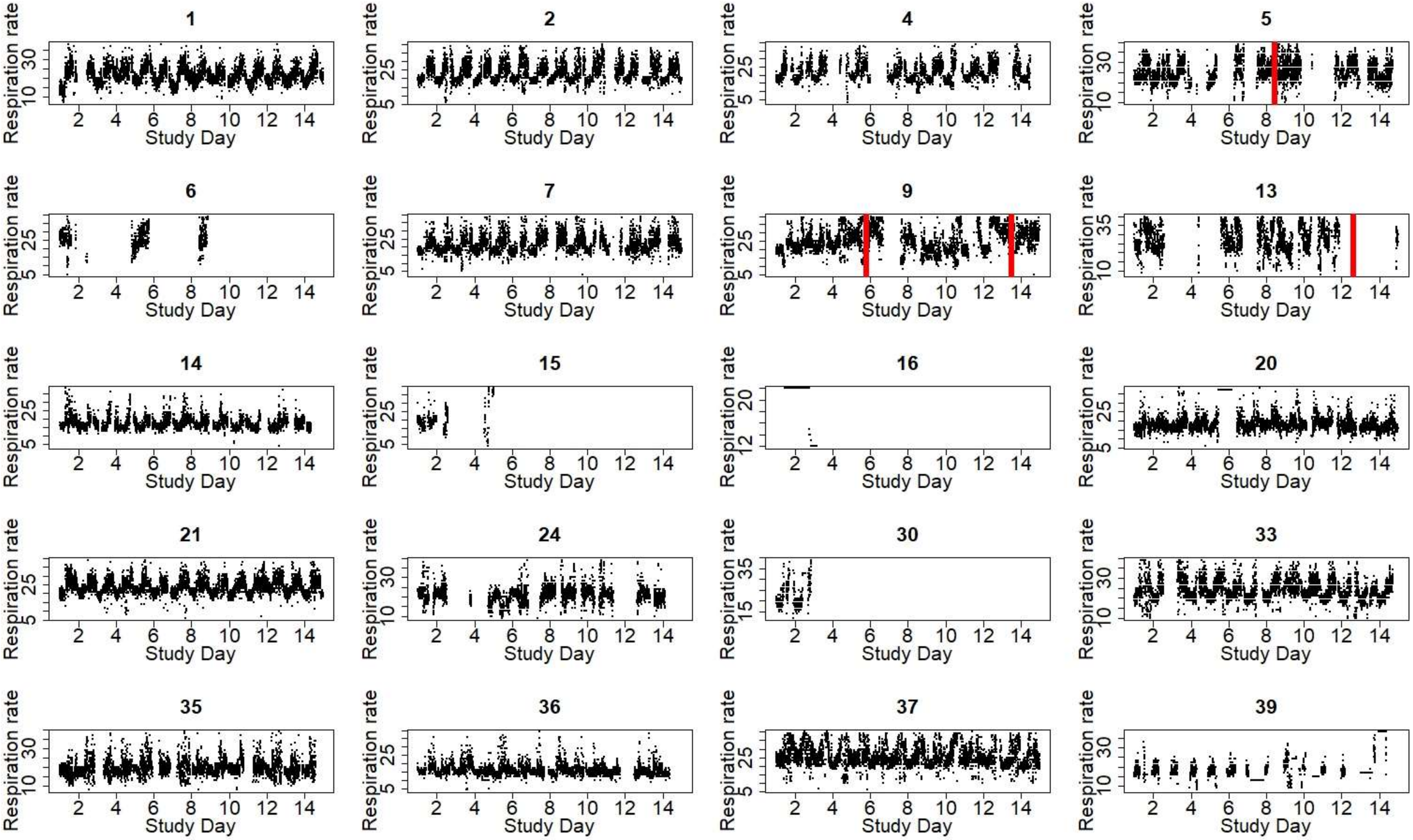

Online Resource Figure S15 – Core temperature, all patients

Recorded core temperature in good quality (score ≥50) in all patients during the 14 days study period. Red lines: event with fever or infection.

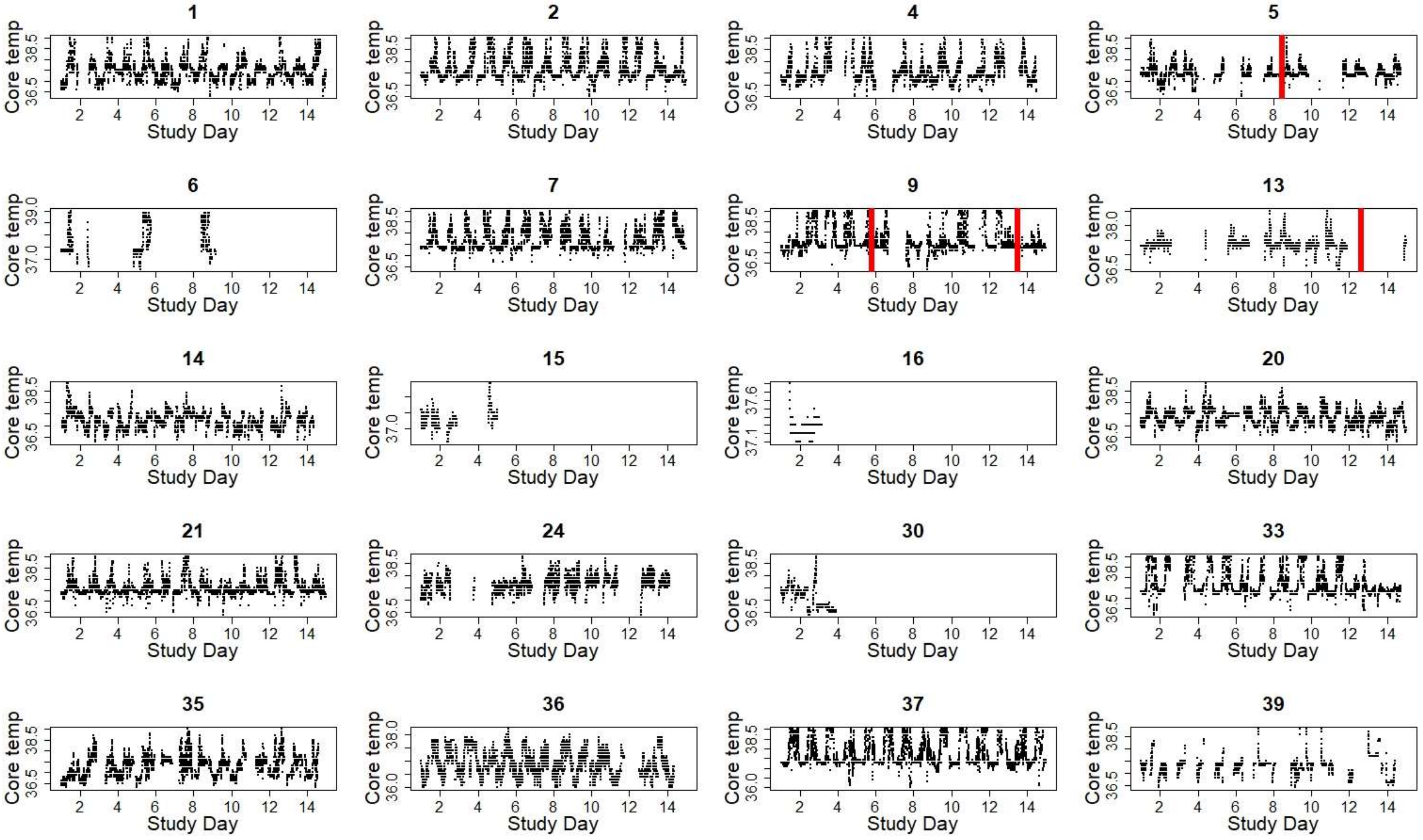

Online Resource Figure S16 – Skin temperature, all patients

Recorded skin temperature in all patients during the 14 days study period. Red lines: event with fever or infection

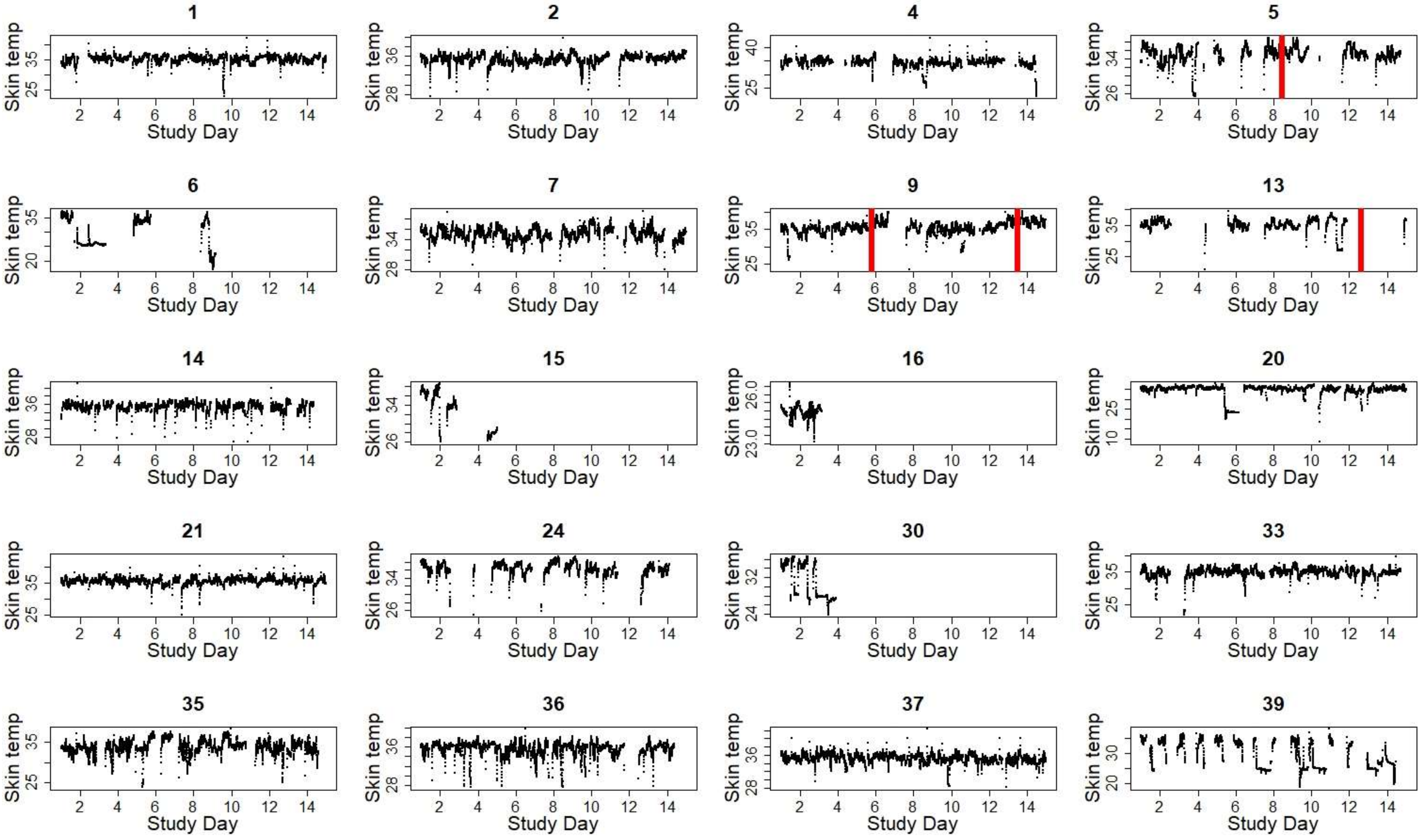

Online Resource Figure S17 – Skin blood perfusion, all patients

Recorded skin blood perfusion in good quality (score ≥50) in all patients during the 14 days study period. Red lines: event with fever or infection

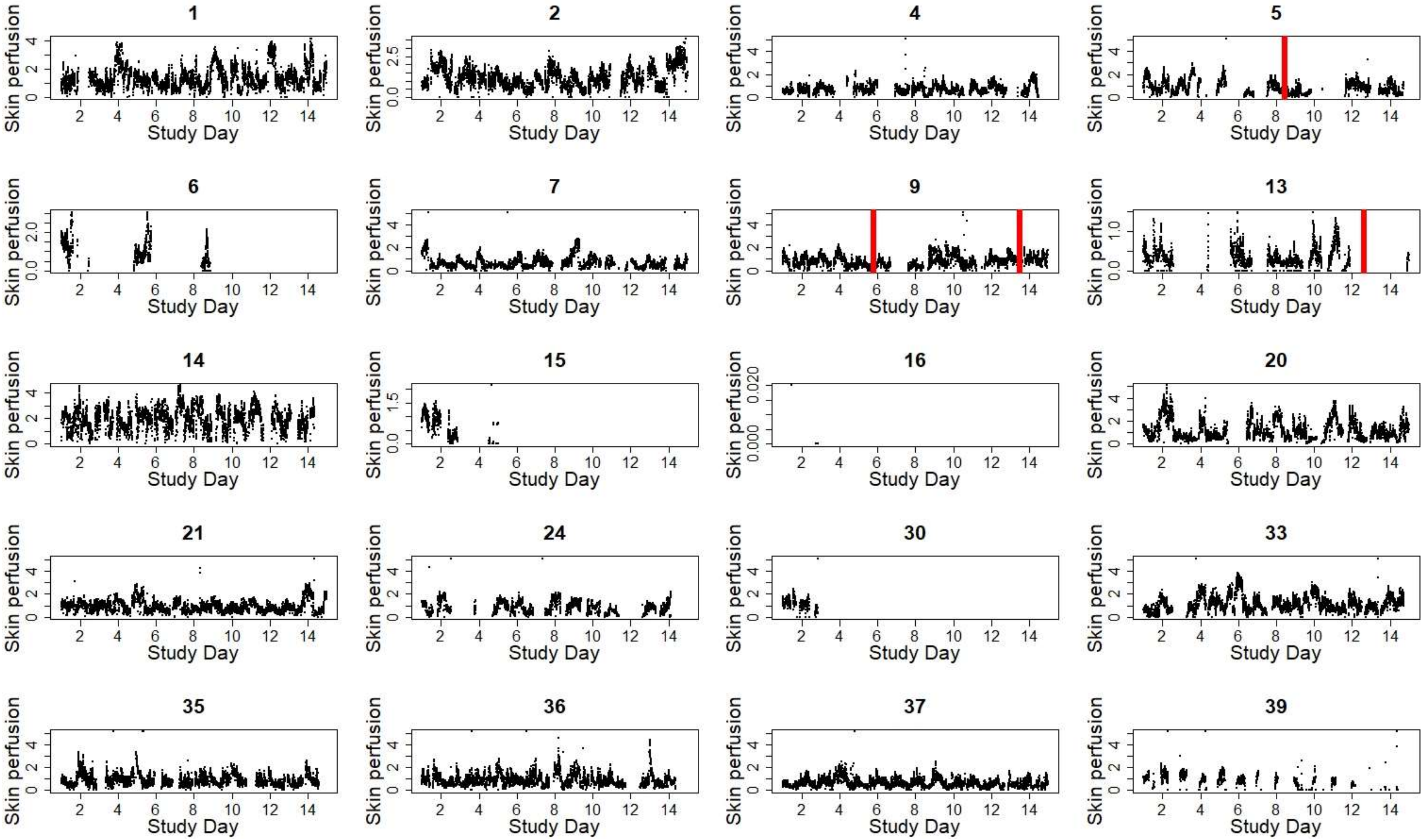

Online Resource Figure S18 – Galvanic skin response, all patients

Recorded galvanic skin response (GSR) in all patients during the 14 days study period. Red lines: event with fever or infection

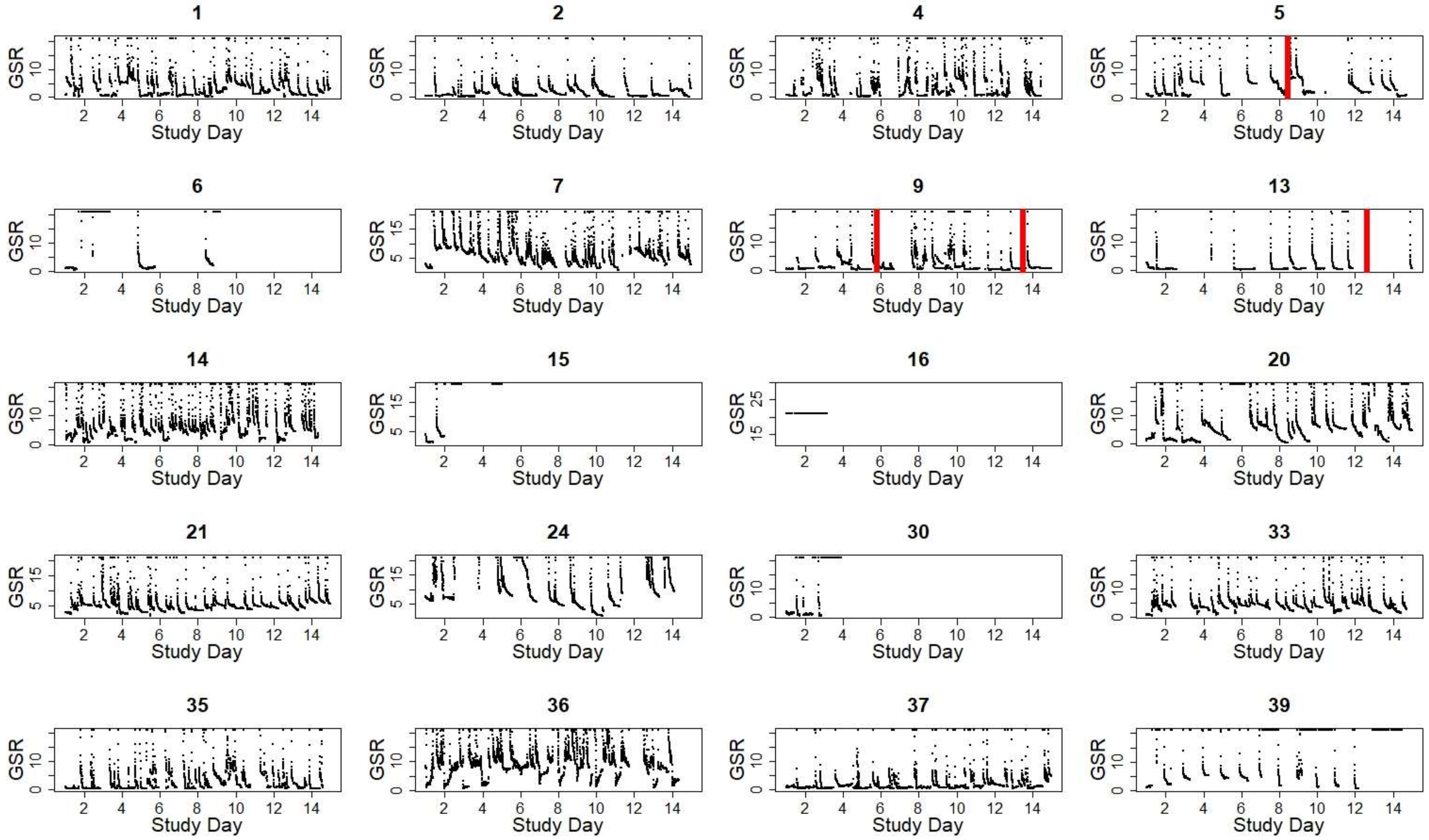

Online Resource Figure S19 – Health score, all patients

Recorded health score in all patients during the 14 days study period. Red lines: event with fever or infection

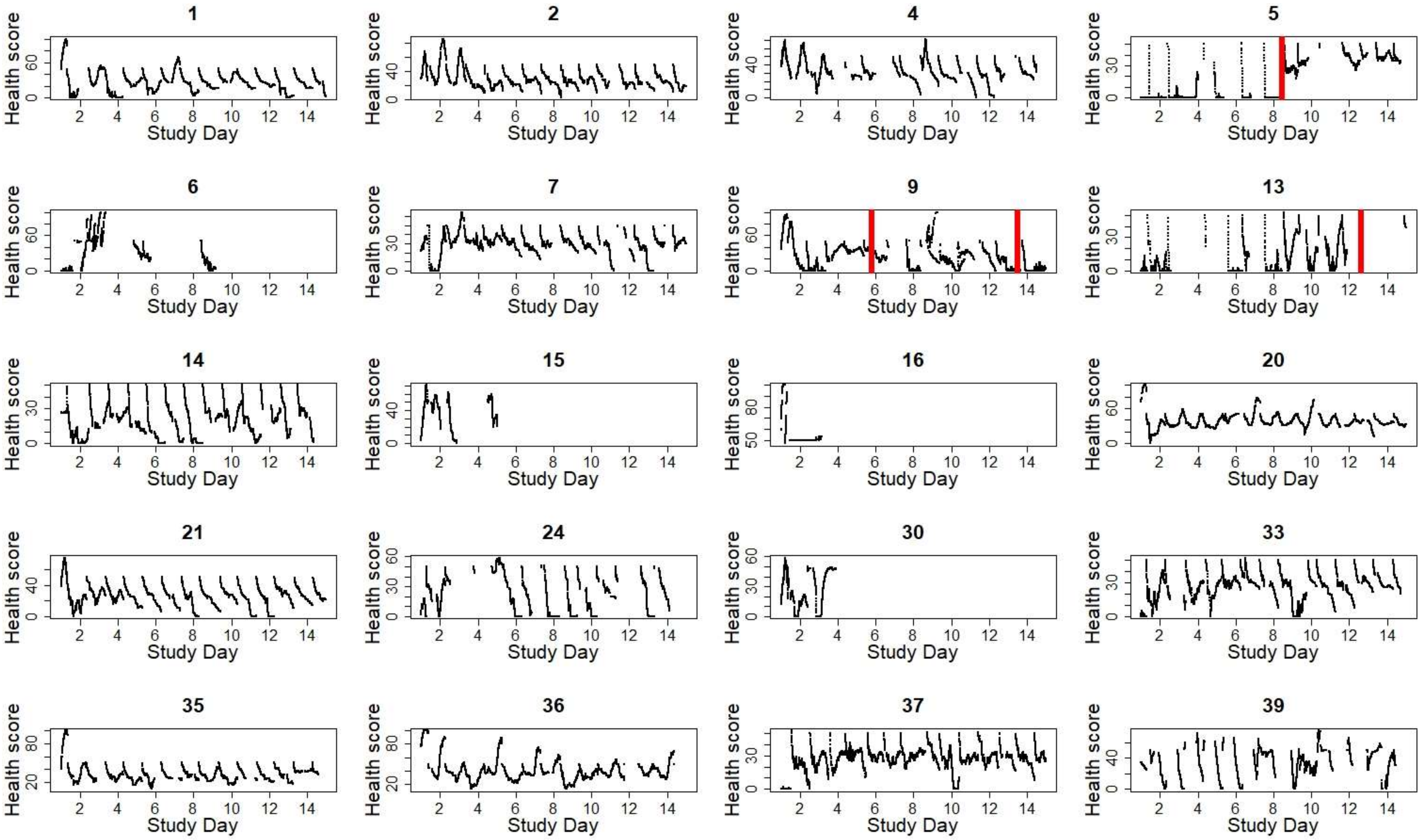

Online Resource Figure S20 – Exploration of heart rate variability preceding and following fever or infection

Recorded heart rate variability in good quality (score ≥50) preceding and following episodes with fever or infection (red line). Green line: mean and 95% confidence interval, blue line: regression line

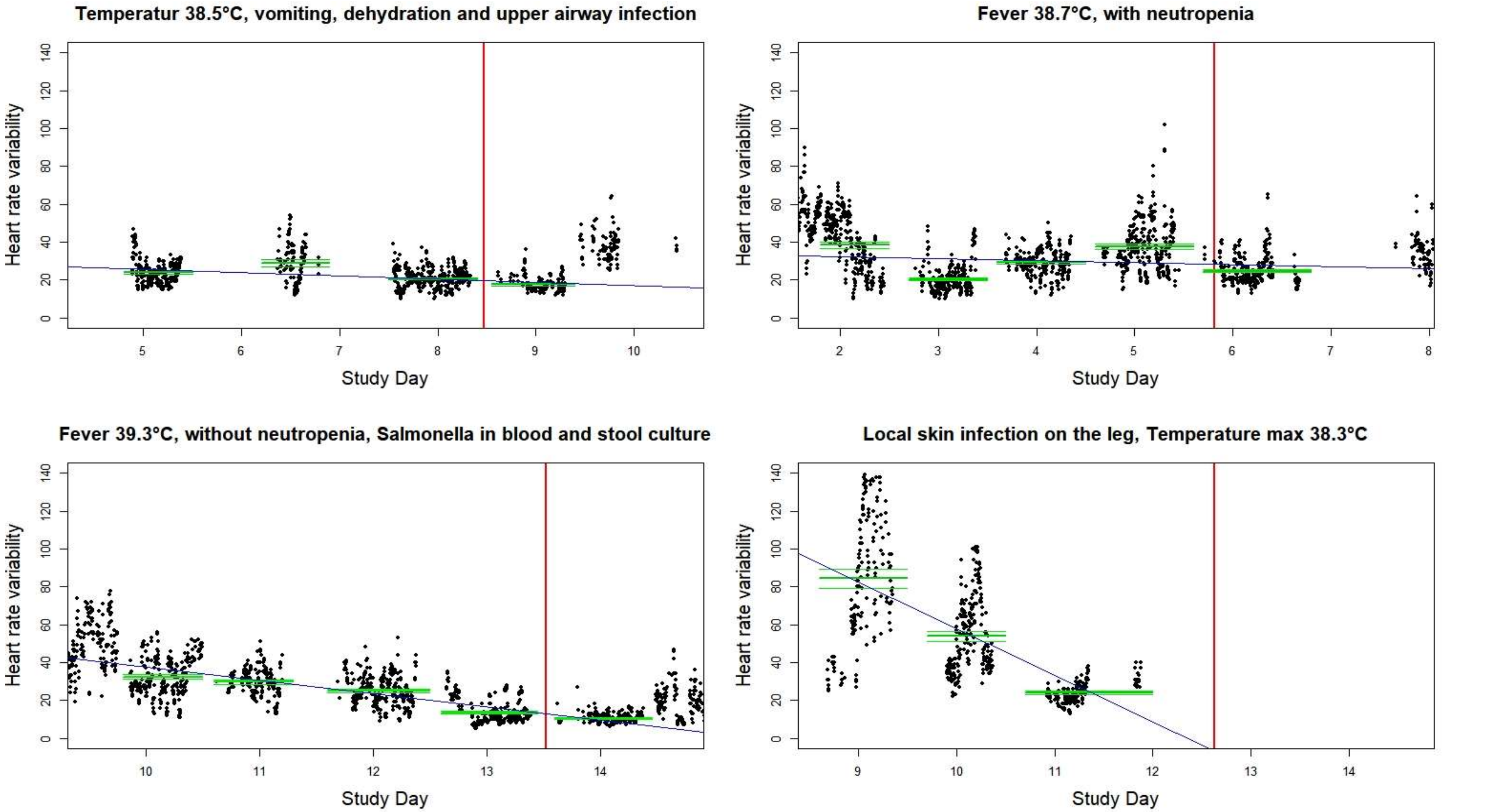

Supplement: Supplementary file 1 — (PDF 3.06 mb) [file 520_2021_6099_MOESM1_ESM.pdf]
